# Expression of Vitamin D Receptor and Vitamin D Receptor Gene Polymorphisms (*BsmI*, *FokI*, and *TaqI*) in Patients with Pterygium

**DOI:** 10.5935/0004-2749.20210032

**Published:** 2021

**Authors:** Şemsettin Bilak, Muhammer Özgür Çevik, İbrahim Halil Erdoğdu, Haydar Bağış

**Affiliations:** 1 Department of Ophthalmology, School of Medicine, Adıyaman University, Adıyaman, Turkey; 2 Department of Medical Genetics, School of Medicine, Adıyaman University, Adıyaman, Turkey; 3 Department of Pathology, School of Medicine, Adnan Menderes University, Aydın, Turkey

**Keywords:** Pterygium, Vitamin D, Polymorphism, genetic, Immunohistochemistry, Pterígio, Vitamina D, Polimorfismo genético, Imunohistoquímica

## Abstract

**Purpose:**

This study aimed to determine the role of vitamin D receptor in the pathogenesis of pterygium. The vitamin D receptor eexpression levels in pterygium tissue, blood vitamin D levels, and frequency of selected vitamin D receptor gene polymorphisms (*BsmI, FokI,* and *TaqI*) were compared between patients with pterygium and healthy participants.

**Methods:**

The study included patients with pterygiumeee (n=50) and healthy volunteers (n=50). The serum vitamin D levels were measured for both groups. Immunohistochemical staining for vitamin D receptor ewas performed on sections obtained from the pterygium and adjacent healthy conjunctival tissues of the same individuals. The genomic existence of vitamin D receptor epolymorphisms (*BsmI, FokI*, and *TaqI*) were analyzed in DNA obtained from venous blood of participants using polymerase chain reaction and restriction fragment length polymorphism methods.

**Results:**

There was no difference found between the serum vitamin D levels of patients with pterygium and healthy controls. However, tissue expression of vitamin D receptor was higher in the pterygium endothelial cells of micro-vessels (p=0.002), subepithelial stromal (p=0.04), and intravascular inflammatory cells (p=0.0001), in comparison with the adjacent healthy conjunctival tissue. Moreover, while the BBtt haplotype was 2-fold higher, the bbTt haplotype was 2.5-fold lower, and the BbTT haplotype was 2.25-fold lower in the control group than in the pterygium group (p<0.001).

**Conclusions:**

Vitamin D serum levels did not differ between the healthy and pterygium groups. Vitamin D receptor expression was increased in the pterygium tissue versus the adjacent healthy tissue. However, vitamin D receptor polymorphism analysis in patients with pterygium did not reveal any significant difference in *BsmI, FokI*, or *TaqI* polymorphisms in comparison with the healthy volunteers.

## Abstract (pt)

**Objetivo:**

Determinar o papel do receptor da vitamina D na patogênese do pterígio. Os níveis de expressão do receptor da vitamina D no tecido do pterígio, os níveis sanguíneos de vitamina D e a frequência de alguns polimorfismos do gene do receptor da vitamina D (*BsmI, FokI* e *TaqI*) foram comparados entre pacientes com pterígio e participantes saudáveis.

**Métodos:**

Foram incluídos pacientes com pterígio (n=50) e voluntários sau dáveis (n=50). Os níveis séricos de vitamina D foram medidos em ambos os grupos. Foi feita uma coloração imuno-histoquímica para o receptor da vitamina D em cortes obtidos do pterígio e dos tecidos conjuntivais saudáveis adjacentes dos mesmos indivíduos. A existência de polimorfismos do receptor da vitamina D (*BsmI, FokI* e *TaqI*) no genoma foi analisada em DNA obtido do sangue venoso dos participantes, usando métodos de Polymerase chain reaction (PCR) e RFLP.

**Resultados:**

Não foi observada nenhuma diferença entre os níveis séricos de vitamina D dos pacientes com pterígio e os dos controles saudáveis. Entretanto, a expressão tissular do receptor da vitamina D foi maior nas células endoteliais dos microvasos do pterígio (p=0,002), nas células estromais sub-epiteliais (p=0,04) e nas células inflamatórias intravasculares (p=0,0001), quando comparada à expressão no tecido conjuntival saudável adjacente. Além disso, embora o haplótipo *BBtt* tenha sido duas vezes mais frequente, o haplótipo *bbTt* foi 2,5 vezes menos frequente e o haplótipo *BbTT* foi 2,25 vezes menos frequente no grupo de controle do que no grupo com pterígio (p<0,001).

**Conclusões:**

Os níveis séricos de vitamina D não apresentaram diferenças entre o grupo de pessoas saudáveis e o com pterígio. A expressão do receptor da vitamina D mostrou-se maior no grupo com pterígio do que no tecido saudável adjacente. Entretanto, a análise dos polimorfismos do receptor da vitamina D nos pacientes com pterígio não revelou qualquer diferença significativa nos polimorfismos *BsmI, FokI* ou *TaqI* em comparação com os voluntários saudáveis.

## INTRODUCTION

Pterygium is the fibrovascular invasive growth of nasal bulbar conjunctiva over the corneal surface. It can cause ocular irritation, visual disturbances, and cos metic problems^(1,2)^. Although many studies have investigated pterygium, its pathophysiology has not been clarified yet. Substantial evidence from epidemiological studies has confirmed that chronic exposure to ultraviolet radiation (UVR) is a significant risk factor for the formation of pterygium^(2-4)^. Other risk factors include chronic inflammation, viral infection, age, dry eye disease, oxidative stress, anti-apoptotic mechanism, growth and angiogenesis, and genetic factors^(1,4)^. Not all individuals sharing the same environmental conditions develop pterygium. This suggests that genetics may play a role in this process, but the underlying mechanisms have not been elucidated yet^(5)^.

It is well established that vitamin D is a multifunctional steroid hormone, and a sufficient serum vitamin D level is essential for ocular surface health^(6)^. Vitamin D has anti-angiogenic, anti-inflammatory, anti-proliferative, and anti-fibrotic effects on the ocular surface. In addition, angiogenesis, inflammation, proliferation, and fibrosis are critical features of the pathogenesis of pterygium^(1,4,6)^. Therefore, vitamin D is expected to play a protective role in the formation of pterygium. In contrast, two studies reported that serum levels of vitamin D were high in patients with pterygium^(7,8)^. These results indicate that the circulating vitamin D may not be sufficiently protective to conjunctival tissue. This is attributed to decreased or less effective forms of expression of vitamin D receptor (VDR) receptors due to polymorphisms. It is known that the effects of vitamin D on tissue depend on the expression and functional status of the VDR in tissue^(9)^. We selected a well-recognized approach of phenotyping. Single-nucleotide polymorphisms are the most common DNA sequence variations in the human genome, and they are a useful method for recognizing gene-associated diseases and predisposition of patients to diseases^(10)^. Moreover, VDR polymorphism is found in several ocular diseases, such as myopia, primary openan gle glaucoma, and dry eye disease^(12-14)^. VDR polymorphism is the most frequent polymorphism in Caucasian populations, which is the population examined in our study^(11)^. The present study aimed to investigate the role of VDR polymorphism and the expression of the VDR receptor in the pathogenesis of pterygium. To our knowledge, this is the first study that investigated VDR polymorphisms in patients with pterygium.

## METHODS

### Study design and population

This cross-sectional study was conducted in the Department of Ophthalmology and Medical Genetics at Adıyaman University School of Medicine (Adıyaman, Turkey). Written informed consent was provided by all participants. The study was performed in accordance with the tenets of the Declaration of Helsinki. The study was approved by the Ethics Committee of Adıyaman University Faculty of Medicine. Fifty patients with pterygium and 50 healthy volunteers (age-and gender-matched) were enrolled in the study. Data collected from all participants included age, sex, ocular pathology, topical medication use, and surgical history. Complete ocular examinations were performed, including visual acuity, biomicroscopy, tonometry, and fundus examination. Patients with ocular pathology, past ocular surgery, and topical medication use were excluded from the study. Pterygium was diagnosed clinically using slit-lamp biomicroscopy; it was defined as a fibrovascular overgrowth of the nasal bulbar conjunctiva over the cornea.

Patients with pterygium underwent primary pterygium excision surgery through the conjunctival autograft technique. This surgical technique requires the re moval of an autograft from an adjacent tissue to the pterygium to cover the area of the excised pterygium. This autograph is generally obtained from the healthy conjunctival tissues at the border of the graft bed at the upper temporal part of the bulbar conjunctiva of the same eye. However, there are always minute amounts of tissue left after trimming. Residual autograft after trimming was used as a healthy control for tissue staining experiments. The excised pterygium tissue formed the pterygium group. For the control group, the residual autograft tissues of the same pterygium patients were used. The tissues were formalin-fixed and paraffin-embedded for immunohistochemical analysis.

Whole blood samples were collected from the antecubital veins of patients with pterygium (pterygium group) and healthy volunteers (control group). Blood sam ples were placed in ethylene diamine tetra-acetic acid (EDTA)-coated vacutainer tubes (BD Medical, Franklin Lakes, NJ, USA) and serum separator gel tubes (BD Medical). For the serum vitamin D analysis, whole blood samples of all the participants were centrifuged (3,500 rpm for 10 min) in serum separator gel tubes containing serum separator gel. The serum vitamin D levels were measured using electrochemiluminescence immunoassay (Hitachi High-Technologies Corporation, Tokyo, Japan) in an automatic electrochemiluminescence analyzer (Roche Diagnostics Co. Ltd., Mannheim, Germany).

### VDR polymorphism analysis

**Figure 1:**
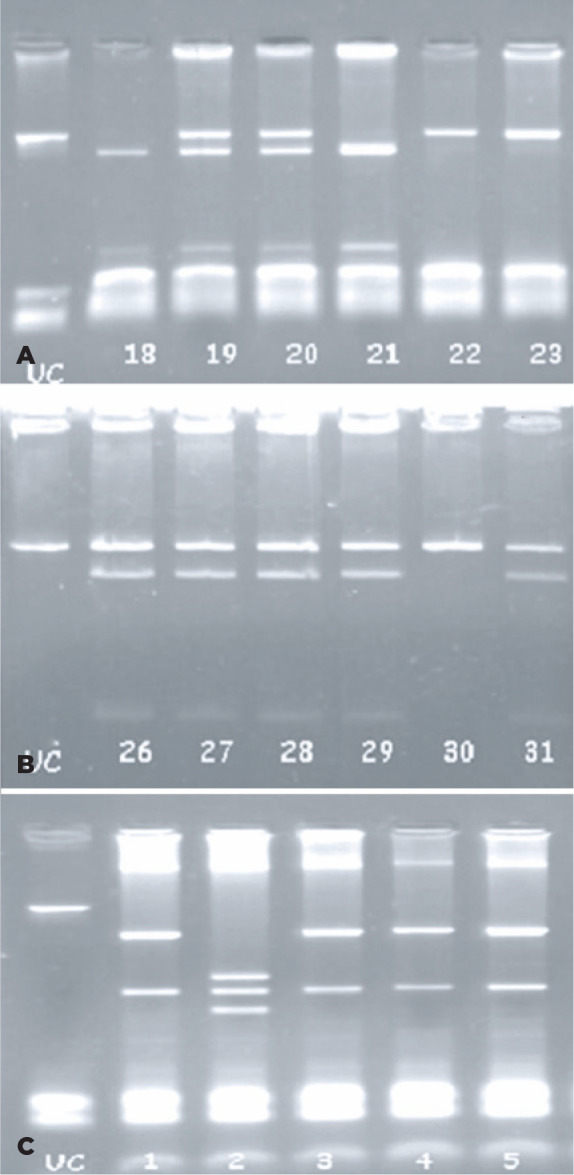
(A) Vitamin D receptor (VDR) BsmI polymorphism restriction fragment length polymorphism (RFLP) genotyping. The samples of the bb genotypes are the 18^th^ and 21^st^. The samples of Bb genotype are 19^th^ and 20^th^. The samples of the BB genotype are the 22^nd^ and 23^rd^. (B) VDR FokI polymorphism RFLP genotyping. The samples of the Ff genotype are the 26^th^, 27^th^, 28^th^, 29^th^, and 31^st^. The sample of the FF genotype is the 30^th^ (C) VDR TaqI polymorphism RFLP genotyping. The samples of the TT genotype are the 1^st^, 3^rd^, 4^th^, and 5^th^. The sample of the tt genotype is the 2^nd^. UC: uncut polymerase chain reaction product.

After collecting whole venous blood from both control and pterygium groups in EDTA-containing tubes, DNA was isolated using the phenol-chloroform method. Haplotypes of the *BsmI* (rs1544410), *FokI* (rs 2228570), and *TaqI* (rs 731236) polymorphisms were analyzed using polymerase chain reaction and restriction fragment length polymorphisms, as previously described (Table 1)^(15)^. Briefly, the VDR polymorphisms of *BsmI, FokI*, and *TaqI* were screened with *BsmI, FokI,* and *TaqI* restriction enzymes, according to the procedure reported in Panierakis et al. with minor modifications ^(15)^. Restriction with endonucleases was visualized under UV after running in a 3% agarose gel electrophoresis (Orange G was used as the dye) in 100-volt potential for 30 min in an electrophoresis cast (Biogen, MA, USA) (Figure 1).

**Table 1:** PCR-RFLP primers for VDR gene polymorphisms (*BsmI, FokI*, and *TaqI*)

SNPs	Primer (5’ → 3’) Forward	Primer (5’ → 3’) Reverse	bp
BsmI	CAA CCA AGA CTACAA GTA CCG CGT CAG TGA	AAC CAG CGG GAAGAG GTC AAG GG	825
FokI	AGC TGG CCCTGG CAC TGA CTC TGC TCT	ATG GAA ACA CCTTGC TTC TTC TCC CTC	265
TaqI	CAG AGC ATGGAC AGG GAG CAA	GCA ACT CCT CATGGC TGA GGT C CTC	740

PCR-RFLP= polymerase chain reaction and restriction fragment length polymorphisms; VDR= vitamin D receptor; SNP= single-nucleotide polymorphism; bp= base pair.

### Immunohistochemical staining

Staining for VDR proteins was performed in the sec tions obtained from the same blocks used for the hem atoxylin-eosin staining. The 4-µm thick sections were prepared from the materials embedded in the paraffin blocks after routine tissue monitoring in 10% neutral-buffered formalin. The sections were placed on positively-charged Poly-L-lysine coated slides (Thermo Fisher Scientific, Waltham, MA, USA) and maintained in a dry oven at 37°C overnight. Subsequently, the sections were deparaffinized with xylol and dehydrated by passing through an ethyl alcohol series. Antigen retrieval was conducted for 40 min at 96°C using a retrieval solution (DAKO 10 mM/L citrate buffer, pH=6) in a DAKO thermostatic bath (PT Link). The sections were allowed to cool to room temperature. The next steps were performed with an automated system (K8000 Envision Flex; DAKO, Glostrup, Denmark) using the streptavidin-biotin-immunoperoxidase method.

Firstly, we applied super-block reagent (Envision FLEX Peroxidase blocking reagent, 40 mL, K8000; DAKO) to the histopathological sections to block endogenous peroxidase activity. Next, the sections were incubated with the Anti-GC primary antibody (cat. no: HPA001526; Atlas Antibodies, Stockholm, Sweden) and the vitamin D solution onto each section to completely cover the tissue for 60 min. The sections were incubated for 10 min with the addition of a secondary antibody (Envision FLEX/HRP; DAKO). The colorant, diaminobenzidine tetrachloride (DAB, K8000; DAKO) was used to demonstrate binding due to immunoreactivity. The sections were counter-stained with hematoxylin. After washing, the sections were passed through the series of decreasing alcohol dilutions, and the sections were closed with lamellae after xylol clearing, dried, and coated with enamel. We used human skin tissue as a positive control to test the effectiveness of staining. While the positive control tissues were stained, normal rabbit serum IgG was used as a negative control instead of a primary antibody. The immunohistochemically stained sections were examined by a clinically-blinded expert pathologist (author: Erdogdu) at 4×, 10×, 20×, and 40**×** magnifications under light microscopy (BX51; Olympus, Tokyo, Japan). The staining in the subepithelial stromal connective tissue, vascular endothelium, and intravascular cells was evaluated. At least 200 cells were counted in areas with the most intense cytoplasmic and nuclear staining (hot spots). The immunostaining results were scored based on the intensity of the staining. The intensity of VDR expression was graded using the following scale: staining ≤5%: “0”; 5-10% staining: “+”; 11-20% staining: “++”; and 20% staining: “+++,” as previously described by Coskun et al.^(16)^.

### Statistical analysis

Statistical analysis was performed using a commercially available statistical software package (SPSS version 15.0 for Windows; SPSS Inc., Chicago, IL, USA). The Kol mogorov-Smirnov test was used to determine the normality of the distribution. The chi-squared test and Student’s t-test were used to evaluate the demographic characteristics of patients. Descriptive statistics (mean, standard deviation, frequency, and percentage) were used to assess the mean and standard deviation of the demographics and clinical parameters of patients. Student’s t-test was used to analyze the serum vitamin D levels of the two groups. The Wilcoxon test was used to analyze the expression of VDR in the pterygium and control tissues. The chi-squared test was used to analyze each allele and the genotype frequencies of the two groups. The polymorphisms of *BsmI, FokI*, and *TaqI* were tested using the Hardy-Weinberg equilibrium. The risk of pterygium association with each allele and genotype was investigated by calculating the odds ratio. The relationships between the polymorphisms of *BsmI, FokI*, and *TaqI* and the demographic variables were analyzed using logistic regression for age and the chi-squared test for sex; p-values of <0.05 denoted statistically significant differences.

## RESULTS

**Figure 2:**
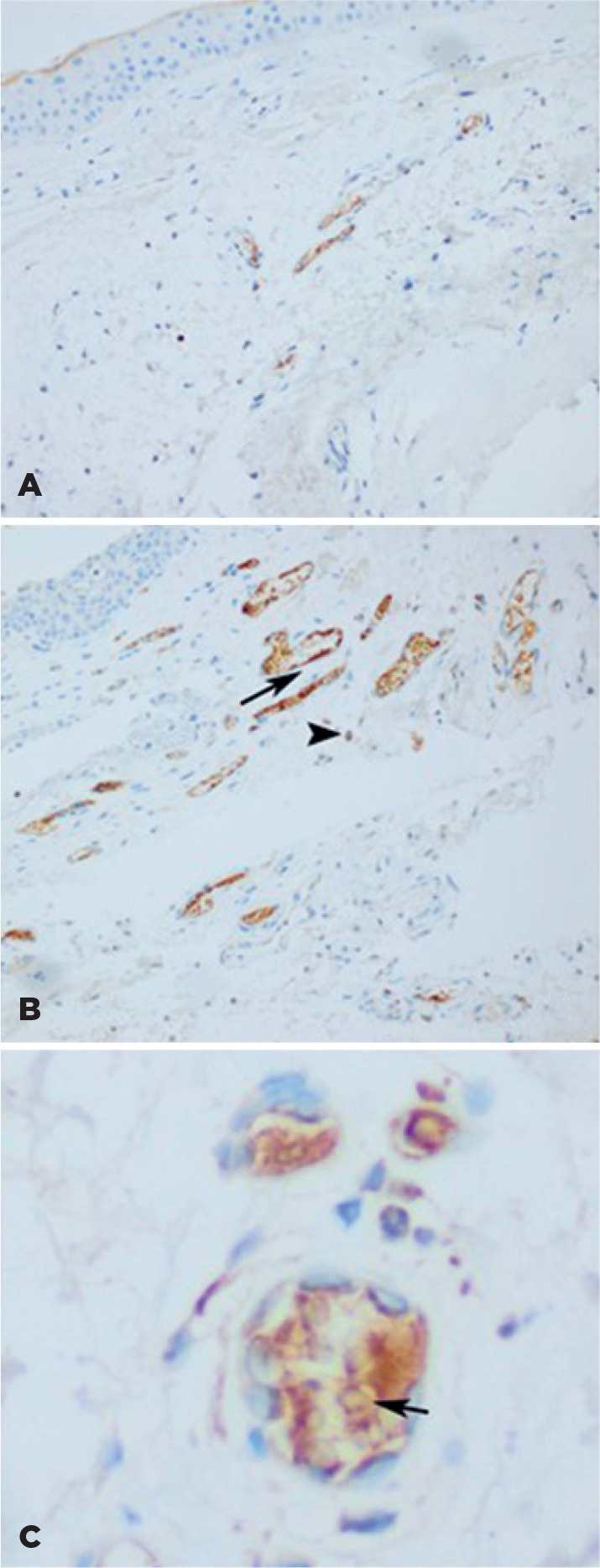
Immunohistochemical expression of vitamin D receptor (VDR) in normal conjunctiva (A) and in pterygium (B, C). Diffuse inflammatory cell infiltration and vascular proliferation were detectable in the connective tissue of pterygium (B). Marked immunostaining of VDR was present in scattered stromal inflammatory cells (arrowhead) and vascular endothelium (arrow) of pterygium (B). VDR showed marked immunoreaction in intravascular inflammatory cells of pterygium (C) (arrow). In normal conjunctiva, only a weak immunostaining for VDR was noticed (A). Original magnification: A, B: 20×; C: 40×.

The age, sex, and serum vitamin D levels did not vary between the two groups (p>0.05) (Table 2). The results of the logistic regression analysis did not show a relationship between the polymorphisms of *BsmI, FokI*, or *TaqI* and age, gender, or serum vitamin D levels (p>0.05) (Table 3). The pathological evaluation showed increased vascular proliferation and inflammatory cell infiltration in the pterygium tissue compared with healthy adjacent tissues. VDR reactive immunostaining was detected in the subepithelial stromal tissue, in the endothelial cells of the subepithelial micro-vessels, and the intravascular cells (Figure 2). The VDR protein expression values for the pterygium and control tissues were 0.47 ± 0.58 ver sus 0.26 ± 0.47 (p=0.04) for the inflammatory cells of the subepithelial stromal tissue, 0.64 ± 0.54 versus 0.32 ± 0.56 (p=0.002) for the vascular endothelium, and 1.92 ± 1.0 versus 1.18 ± 0.99 (p=0.0001) for the intravascular inflammatory cells, respectively (Table 4).

**Table 2:** Characteristics of the pterygium and control groups

Characteristic	Pterygium group	Control group	p-value
Sex (f/m)	29/21	26/24	0.55
Age	51.26 ± 13.27	51.20 ± 13.90	0.98
Vitamin D (ng/mL)	11.66 ± 5.63	10.40 ± 6.30	0.29

f/m= female/male; ng/mL= nanograms per milliliter.

**Table 3:** Logistic regression analysis of the polymorphisms of *BsmI, FokI*, or *TaqI* and age, gender, or serum vitamin D levels

	β	p-value	95% Cl
Bsml			
Age (years)	-0.02	0.26	0.95-1.01
Vitamin D (ng/mL)	0.02	0.60	0.94-1.12
Sex (f/m)	-0.53	0.28	0.22-1.55
Fokl			
Age (years)	0.00	0.86	0.94-1.05
Vitamin D (ng/mL)	0.03	0.63	0.90-1.19
Sex (f/m)	0.77	0.37	0.40-11.45
Taql			
Age (years)	-0.07	0.06	0.87-1.00
Vitamin D (ng/mL)	-0.03	0.66	0.83-1.13
Sex (f/m)	0.06	0.95	0.14-7.85

CI= confidence interval; f/m= female/male; ng/mL= nanograms per milliliter.

**Table 4:** VDR immunostaining results

VDR immunostaining location	Pterygium group	Control group	p-value
Inflammatory cells of subepithelial stroma	0.47 ± 0.58	0.26 ± 0.47	0.04
Endothelial cells of subepithelial micro-vessels	0.64 ± 0.54	0.32 ± 0.56	0.002
Intravascular inflammatory cells	1.92 ± 1.0	1.18 ± 0.99	0.0001

VDR= vitamin D receptor.

The allele frequencies and genotype distributions of the three polymorphisms of interest are presented in table 5. The allelic and genotype frequencies did not reveal significant differences between the pterygium and control groups. All three VDR polymorphisms (*BsmI, FokI*, and *TaqI*) were consistent with the Hardy-Weinberg equilibrium in both groups (Table 5). The most frequent haplotypes were BbTtFF (20%) and BbTt (36%) for the pterygium group, and BbTtFF (26%) and bbTT (46%) for the control group. The risk analysis of the haplotypes with the odds ratio showed that the number of BBtt haplotypes increased, whereas that of bbTt and BbTT haplotypes decreased in both groups. However, the increase and decrease observed in the control group was 2-fold higher than those observed in the pterygium group (p<0.001).

**Table 5:** Genotype and allele distribution between the pterygium and control groups

SNP	Pterygium η (%)		Control η (%)	Total η (%)	p-value	OR	95% Cl
Bsml	**BB**	11 (22.0%)	5 (10.0%)	16 (16.0%)	0.21^*^		
	**Bb**	22 (44.0%)	22 (44.0%)	44 (44.0%)			
	**bb**	17(34.0%)	23 (46.0%)	40 (40.0%)			
	**B**	44 (44.0%)	32 (32.0%)	76 (38.0%)	0.08^**^	1.28	0.98-1.68
	**b**	56 (56.0%)	68 (68.0%)	124 (62.0%)			
Taql	**TT**	21 (42.0%)	25 (50.0%)	46 (46.0%)	0.72^*^		
	**Tt**	24 (48.0%)	21 (42.0%)	45 (45.0%)			
	**tt**	5 (10.0%)	4 (8.0%)	9 (9.0%)			
	**T**	66 (66.0%)	71 (71.0%)	137(69.0%)	0.45^**^	0.89	0.67-1.19
	**t**	34 (34.0%)	29 (29.0%)	63 (31.0%)			
Fokl	**FF**	26 (52.0%)	26 (52.0%)	52 (52.0%)	1.00^*^		
	**Ff**	20 (40.0%)	20 (40.0%)	40 (40.0%)			
	**ff**	4 (8.0%)	4 (8.0%)	8 (8.0%)			
	**F**	72 (72.0%)	72 (72.0%)	144 (72.0%)	1.00^**^	1.00	0.73-1.36
	**f**	28 (28.0%)	28 (28.0%)	56 (28.0%)			

* p-value= genotype distribution.

** p-value= allelic distribution; OR and 95% CI is for the allelic distribution.

CI= confidence interval; OR= odds ratio; SNP= single-nucleotide polymorphism.

## DISCUSSION

There is a close relationship between the health of the ocular surface and vitamin D levels. Pterygium is characterized by chronic inflammation and angiogenesis on the ocular surface^(1,2,17)^. Topical administration of vitamin D inhibits ocular surface inflammation and corneal neovascularization^(18)^. Moreover, human corneal limbal epithelial cells and scleral fibroblasts contain both VDR and vitamin D metabolizing enzymes. These can synthesize, activate, and regulate vitamin D levels^(2,3)^. The limbal corneal epithelium acts as a protective barrier against conjunctival invasion, and alteration of this barrier leads to the formation of pterygium^(19)^. UVR from the sun is likely to be the major contributing factor to pterygium formation, but it is also the primary inducer of human vitamin D synthesis. Lin et al. found that corneal limbal epithelial cells were able to *de novo* produce vitamin D in a culture after exposure to UVR^(20)^.

Regulation of the VDR level is an essential mechanism for modulating the response of target tissues to vitamin D. However, the expression of VDR is regulated in a tissue-specific manner^(21)^. Maxia et al. studied the immunolocalization of VDR in ophthalmic pterygium. They found a significant difference in comparison with normal conjunctiva obtained from healthy volunteers. The pterygium samples mainly showed nuclear VDR staining, while the control samples exhibited cytoplasmic staining^(22)^. They hypothesized that pterygium resembles benign neoplastic disorders, so that the nuclear immunolocalization of VDR may represent an alternative nuclear pathway that is related to anti-proliferative and anti-inflammatory effects via the regulation of gene expression. Our study is different since we used healthy conjunctiva obtained from the same eye in the control group, and we did not make an immunolocalization distinction of the VDR. Moreover, Maxia et al. mainly focused on VDR expression in the epithelial layers, while we mainly focused on the subepithelial stromal layers. The scattered light from the inferior or lateral part of the orbital rim is focused 20-fold higher on the nasal limbus where the pterygium primarily occurs^(4)^. We found an increase in VDR expression in the pterygium tissue in comparison with the control tissue from the upper bulbar conjunctiva of the same eye. This suggests that chronic exposure of the conjunctival tissue to UV may cause the up-regulation of VDR expression.

There is a marked increase in the angiogenesis and overexpression of vascular endothelial growth factor in pterygium tissue^(17)^. Vitamin D plays a role in the inhibition of vascular endothelial growth factor-induced endothelial cell differentiation and proliferation^(23)^. We detected a significant increase in VDR expression in the endothelial cells of the subepithelial micro-vessels. Since the anti-angiogenic effect of vitamin D is well-defined in the literature^(23,24)^, we speculate that vitamin D may also have a regulatory effect on angiogenesis by increasing VDR expression in the endothelium. The number of inflammatory cells indicates the severity of the inflammation. Golu et al. found diffuse infiltration of T-lymphocytes, B-lymphocytes, and macrophages in the subepithelial connective tissue in patients with pterygium^(25)^. Pterygium tissue has been reported to have increased levels of inflammatory markers, such as intercellular adhesion molecule-1, vascular cell adhesion molecule-1, and human leukocyte antigen^(26)^. The findings reported in these studies support the role of cellular immunity in the formation of pterygium. However, the number of chronic inflammatory cells is independent of the level of UVR exposure, suggesting the possible presence of an intrinsic mechanism in the regulation of inflammation^(27)^. We found increased VDR expression in the inflammatory cells of the subepithelial stromal tissue and intravascular inflammatory cells. These results are consistent with findings reported in the literature, and may provide evidence for the anti-inflammatory function of vitamin D in pterygium through the expression of VDR in inflammatory cells^(22,25,27)^.

Data regarding VDR polymorphisms in patients with pterygium are limited in the existing literature. Dry eye disease is a risk factor for the formation of pterygium; however, this condition can also cause ocular surface instability and dry eye disease ^(28)^. Hallak et al. studied VDR polymorphism in dry eye disease; while they found a marginal significance in the distributions of *FokI* and *ApaI*, there was no significance observed in *BsmI* and *TaqI*. They found that the study group had a higher minor homozygote genotype (ff) and a minor (f) allele for *FokI* than the control group; the study group also had a higher minor homozygote genotype and a minor allele for *ApaI* in comparison with the control group^(14)^. We did not find any association between the genotypes or allele frequencies (p>0.05). However, we found that the increase in the BBtt haplotype was 2-fold higher, the decrease in the bbTt haplotype was 2.5-fold lower, and the decrease in the BbTT haplotype was 2.25-fold lower in the control group than the pterygium group (p<0.001). Therefore, one can speculate that an increase in the BBtt haplotype and a decrease in the bbTt and BbTT haplotypes play a protective role in the formation of pterygium.

Pterygium and histopathologic changes in the skin related to UVR exposure have similar properties. Pterygium has similar characteristics to those of neoplasms, including cell proliferation, invasion, and recurrence after resection^(1)^. UVR is the major environmental risk factor in skin melanoma and keratinocyte cancers^(29,30)^. Von Schuckmann et al. investigated the association between vitamin D pathway gene polymorphisms and keratinocyte cancers; they found a lower squamous cell carcinoma risk in *BsmI* recessive genotypes^(29)^. In our study, we did not find any association in the genotype distributions between the two groups. Li et al. studied the haplotypes of the VDR gene to evaluate the risk of cutaneous melanoma; they found that VDR polymorphisms directly affect the risk of cutaneous melanoma^(30)^. They showed that the Tttt/BbBB/Ffff haplotypes and Tttt/BbBB/FF haplotypes were also associated with reduced risk, whereas the TT/BbBB/Ffff haplotypes was associated with increased risk. Our study showed that an increase in the BBtt haplotype and a decrease in the bbTt/BbTT haplotypes were associated with decreased risk of pterygium.

To the best of our knowledge, this is the first study to investigate the VDR polymorphisms (*BsmI, FokI*, and *TaqI*) and the VDR expression level in pterygium tissue. Our results showed a possible role of vitamin D in inflammation and angiogenesis in the formation of pterygium. VDR haplotypes and up-regulation in pterygium tissue can play a role in the pathogenesis of this disease. However, further research is warranted to verify this claim.
